# Time to ECG diagnosis delays inter-hospital transfer to revascularization in STEMI patients presenting to a regional emergency department: a five-year audit

**DOI:** 10.1007/s11845-024-03705-6

**Published:** 2024-05-15

**Authors:** Jonathan Shpigelman, Anastasia Proshkina, Marin Roman, Ken Maleady, Ivan Casserly, Gavin Blake, Patrick O’Boyle, Lavanya Saiva, Edward Keelan, James O’Neill, Michael Daly

**Affiliations:** 1 School of Medicine, Royal College of Surgeons in Ireland, Dublin, Ireland; 2 Department of Cardiology, Connolly Hospital Blanchardstown, Dublin, Ireland; 3 Department of Cardiology, Mater Misericordiae University Hospital, Dublin, Ireland

**Keywords:** Door-to-balloon, Myocardial infarction, Percutaneous coronary intervention, ST-segment elevation

## Abstract

**Background:**

Reducing the door-to-balloon time (D2BT) in ST-elevation myocardial infarction (STEMI) patients maximizes myocardial salvage and mitigates morbidity/mortality.

**Aims:**

To assess the D2BT in STEMI patients requiring inter-hospital transfer for revascularization and identify any potential causes of delay.

**Methods:**

Consecutive patients presenting to the Connolly Hospital Blanchardstown (CHB) emergency department (ED) who were transferred to the Mater Misericordiae University Hospital in Dublin for primary percutaneous coronary intervention from January 2018 to October 2022 were identified in a regional database and their D2BTs calculated. D2BTs were further sub-categorized into key intervals to identify any potential causes of delay.

**Results:**

A total of 90 patients were included for analysis, with a median D2BT of 117.5 min (interquartile range [IQR]: 99.3–170.8 min) and 52.5% of patients achieving the ≤ 120 min target. Despite being the shortest interval considered, the time from arrival at the CHB ED to diagnostic electrocardiogram (ECG) was a substantial contributor to the overall delay to revascularization given its wide variability (median: 18.0 min; IQR: 9.0–46.8 min), with only 28.8% of patients achieving the ≤ 10 min target.

**Conclusions:**

Nearly half of the patients studied failed to achieve the overall target D2BT for revascularization. The time from arrival at the CHB ED to diagnostic ECG was identified as a substantial contributor to this failure, with a median time almost twice that of the target and a quarter of all patients spending longer than 46.8 min. These findings highlight a need to improve the implementation of ECG triage and interpretation in the ED.

## Background

ST-elevation myocardial infarction (STEMI) is a cardiac emergency caused by the occlusion of an epicardial coronary artery, typically as the result of atherosclerotic plaque rupture and thrombus formation [1]. The guideline-directed treatment for STEMI is primary percutaneous coronary intervention (pPCI), a minimally invasive procedure involving balloon angioplasty usually with stent implantation [2]. Given that prolonged coronary occlusion correlates with increased infarct size and decreased myocardial salvage [3], timely reperfusion is critical to improving patient outcomes. The door-to-balloon time (D2BT), i.e., the time between first medical contact and coronary reperfusion, is a key institutional quality metric with an established relationship to mortality [4, 5]. Accordingly, current guidelines set a target D2BT at ≤ 90 min for PCI-capable hospitals and ≤ 120 min for non-PCI-capable hospitals that must initiate inter-hospital transfer for definitive management [2]. Despite advances in acute cardiac care, achieving these performance goals remains a challenge, especially for non-PCI-capable hospitals, due to operational complexities [6]. Hence, ensuring robust regional transfer networks and efficient transfer protocols are integral to minimizing D2BTs and optimizing patient outcomes. In this study, we investigated the D2BTs for STEMI patients presenting to the Connolly Hospital Blanchardstown (CHB) emergency department (ED) who required inter-hospital transfer to the Mater Misericordiae University Hospital (MMUH) in Dublin for coronary revascularization via pPCI.

## Methods

### Hospital sites

CHB is a non-PCI-capable teaching hospital within the Royal College of Surgeons in Ireland (RCSI) Hospital Group that serves a large catchment area including West Dublin, North Kildare, and County Meath; its local PCI centre is the MMUH, a tertiary academic centre within the Ireland East Hospital Group that is one of two dedicated centres offering a 24/7 pPCI service to the Greater Dublin region.

### Study population

Consecutive adult patients, i.e., ≥ 18 years, who either self-presented or arrived by ambulance to CHB’s ED and were then transferred to the MMUH for pPCI from January 2018 to October 2022 were identified in the MMUH pPCI registry and retrospectively studied.

### Data collection

Clinical characteristics and treatment timelines for the included patients were downloaded from the MMUH’s electronic pPCI registry. D2BTs (arrival at CHB to first intra-coronary balloon inflation at the MMUH) in those with angiographically-confirmed acute coronary occlusions were sub-categorized into: Interval 1 = arrival at CHB to diagnostic electrocardiogram (ECG); Interval 2 = diagnostic ECG to arrival at the MMUH; and Interval 3 = arrival at the MMUH to first intra-coronary balloon inflation. The respective targets for D2BT and Interval 1 were defined as ≤ 120 min and ≤ 10 min, consistent with current guideline recommendations [2, 7]. However, the targets for Intervals 2 and 3 are not standardized and were defined by local expert consensus, i.e., ≤ 90 and ≤ 20 min, respectively, while ensuring the sum of all interval targets equals that of the total D2BT target. Patients were excluded from analysis if their D2BT was > 12 h or if Interval 1 was ≤ 0 min, the latter indicating either a diagnostic ECG that was obtained prior to arrival at CHB or an erroneous entry into the MMUH pPCI registry. For patients with Interval 1 > 150 min, suggesting an atypical diagnostic delay, e.g., evolving STEMI, a partial exclusion strategy was implemented: D2BT and Interval 1 were omitted to control for outlier effects, while Intervals 2 and 3 were retained.

### Statistical analysis

Categorical data are reported as number (percentage) while continuous data are reported as mean ± standard deviation (SD) or median (interquartile range [IQR]). Given the non-Gaussian distribution of the data as assessed by the Shapiro–Wilk test, comparisons of continuous data were performed using the non-parametric Kruskal–Wallis test. IQR/median ratios were used to compare relative dispersions between data, analogous to the coefficient of variation for Gaussian distributions [8]. Statistical significance was defined as a two-tailed *p* < 0.05. All analyses were performed using GraphPad Prism version 9.5.1 (GraphPad Software, San Diego, California USA).

## Results

### Study population and characteristics

**Fig. 1:**
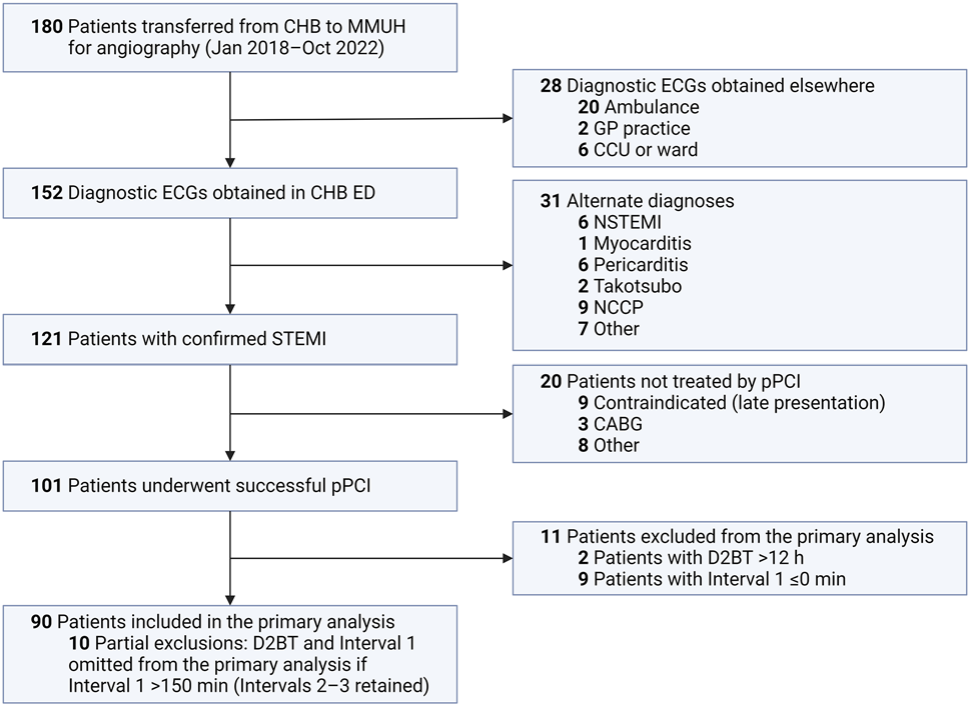
Flowchart for patient selection and inclusion. CABG = coronary artery bypass grafting; CCU = cardiac care unit; GP = general practitioner; NCCP = non-cardiac chest pain; NSTEMI = non-ST-elevation myocardial infarction. Created with BioRender.com

The patient selection method is summarized in Fig. 1. Between January 2018 and October 2022, 180 patients were transferred from CHB to the MMUH for pPCI. Of these, 152 had a diagnostic ECG undertaken in the CHB ED, while the remaining 28 patients, who obtained a diagnostic ECG in other settings (most commonly in an ambulance), were excluded. Of those with a diagnostic ECG obtained in the CHB ED, 121 had acute coronary occlusion confirmed at angiography, 101 of whom underwent pPCI. The most commonly documented reason for not undergoing pPCI was a late clinical presentation. Eleven (11) patients with either D2BT > 12 h or Interval 1 ≤ 0 min were excluded from the primary analysis. Lastly, 10 patients with Interval 1 > 150 min were partially excluded, with their Intervals 2 and 3 retained. Of these, five had documented evolving STEMI that could account for the prolongations of Interval 1; however, for the remaining five patients, no specific reasons were documented to explain these delays.

The baseline characteristics of those included in the primary analysis (*n* = 90) are summarized in Table 1. Overall, the average age was 59.1 ± 12.4 years; 24.4% were female, 7.8% had a history of myocardial infarction, 15.6% had a history of angina, 13.3% had previously undergone PCI, and 34.4% were current cigarette smokers.

**Table 1:** Baseline characteristics of patients included in the primary analysis

**Characteristic**	**Total** ***n= 90***
Demographics	
Age, years	59.1 ± 12.4
Female sex	22 (24.4)
Previous revascularization	
PCI	12 (13.3)
CABG	1 (1.1)
Medical history	
Previous MI	7 (7.8)
Previous angina	14 (15.6)
Previous PVD	3 (3.3)
Previous CVD	3 (3.3)
Diabetes	8 (8.9)
Hypertension	33 (36.7)
Hypercholesterolemia	21 (23.3)
Social history	
Current smoker	31 (34.4)
Former smoker	21 (23.3)

Continuous data are presented as mean ± SD, categorical data are presented as number (percentage)

*CABG* coronary artery bypass grafting, *CVD* cerebrovascular disease, *PVD* peripheral vascular disease

### D2BT and sub-interval analysis

**Fig. 2:**
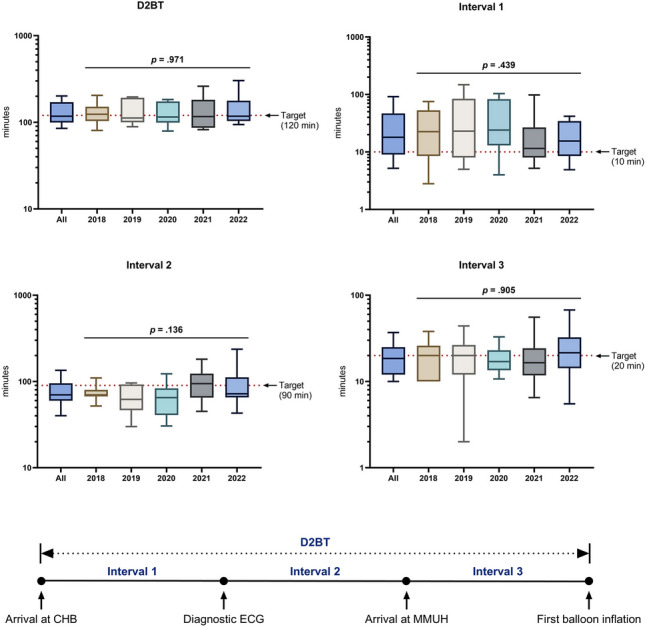
Box and whisker plots for D2BT and key intervals. Whiskers represent 10-90th percentile. Statistical comparisons between years were performed using the non-parametric Kruskal–Wallis test

The distributions for D2BT and Intervals 1–3 are summarized in Fig. 2. Overall, the median D2BT (in minutes) was 117.5 (IQR: 99.3–170.8), with 52.5% of patients achieving the target; no significant differences were observed between the years studied (*p* = 0.971). For Intervals 1–3, the median values were 18.0 (IQR: 9.0–46.8), 70.0 (IQR: 59.8–95.3), and 18.5 (IQR: 12.0–25.0), with 28.8%, 72.2%, and 55.6% of patients meeting their respective targets; again, no significant differences were observed between the years studied. The IQR/median ratios, i.e., the relative dispersions, for Intervals 1–3 were 2.1, 0.5, and 0.7, respectively, indicating that Interval 1 exhibited the greatest variability.

## Discussion

In this five-year retrospective audit of patients presenting to a regional ED with STEMI requiring inter-hospital transfer for pPCI, the median D2BT was 117.5 min, which is just within the guideline-directed target of ≤ 120 min for non-PCI-capable hospitals. However, given that the median is a measure of central tendency, this necessarily means that a substantial proportion (47%) of our patients did not meet the international target, underscoring a need to optimize the acute STEMI pathway. In our analysis, the total D2BT was sub-categorized into three intervals to better resolve any causes of delay. Interval 1 (door-to-diagnostic ECG) was the only interval with a median value (18.0 min) above its target (≤ 10 min), with only 28.8% of patients achieving it. Despite being only 8.0 min above the target, considerable dispersion in the data was observed, with a quarter of all patients spending longer than 46.8 min in the ED before their diagnostic ECGs were obtained. As suggested by their median-normalized IQRs, the variability of Interval 1 was four times greater than that of Interval 2 (diagnostic ECG to arrival at PCI centre) and three times greater than that of Interval 3 (arrival at PCI centre to balloon inflation). Therefore, to improve the timeliness and consistency of Interval 1 at non-PCI-capable hospitals, we suggest that efforts be made to ensure the rapid triage of patients presenting to the ED with ischaemic-type symptoms while maintaining a low threshold of suspicion for acute coronary syndromes (ACS) in those presenting with atypical symptoms and a high-risk profile. In this regard, a forcing function implemented at patient registration could be beneficial, i.e., one requiring the documentation of chest pain and/or other symptoms suggestive of ACS, the presence of which would mandate automatic prioritization to ECG.

In contrast to Interval 1, the medians for Intervals 2 and 3 were within their targets and exhibited far less variability; yet a proportion of patients still failed to achieve these targets. Within the scope of the ED, the primary means of reducing Interval 2 is ensuring prompt ECG interpretation and the activation of emergency medical services (EMS) immediately upon making a diagnosis. Therefore, in patients triaged as potential ACS, it is critical to have a senior clinician available to rapidly interpret the ECG and make a timely decision on management. In circumstances where an on-site cardiologist is not immediately available, ECG data should be rapidly transmitted to the designated PCI centre for external review. Moreover, we hypothesize that delays in ECG diagnosis are often the result of an inappropriate reliance on quantitative serum biomarker testing, i.e., high-sensitivity troponin-T, when the initial diagnosis should be based on clinical presentation and ECG findings alone. To address this, clear protocols should be implemented to ensure prompt senior review when the clinical presentation and ECG findings suggest an ACS, before confirmatory biomarker testing is available. In cases where the ECG findings and/or the symptomatic presentation are equivocal, troponin testing should be considered as an adjunct to clinical diagnosis. Apart from these considerations, Interval 2 is largely dependent on patient transfer times. Given the unavoidable delays associated with EMS due to resource limitations and varying local conditions, there is an argument to be made for having a dedicated ambulance at regional non-PCI-capable centres, although the benefits for STEMI management and other critical transfer scenarios would need to be carefully weighed against the obvious resource implications. Regarding Interval 3, given that the MMUH already employs a single-call activation framework [9] for their cardiac catheterization laboratory (CCL), there is limited opportunity to improve its efficiency. Nevertheless, ensuring efficient communication between EMS and the PCI centre and/or real-time tracking of patient transfers should be prioritized to enable seamless transitions of care.

### Standardized reporting at the transferring hospital

Our analysis relied on data collected by the MMUH pPCI registry, which included only a few key timepoints for the intervals comprising the total time spent at the transferring non-PCI-capable hospital. To obtain a more comprehensive understanding of the factors contributing to any delays in achieving the recommended D2BT, it would be beneficial for the transferring hospital to implement standardized reporting procedures for patients with suspected ACS to collect additional data points including the times of symptom onset, triage, first ECG, first positive ECG, EMS and CCL activation, and patient handover to EMS. This would facilitate more accurate internal review and the provision of data-driven feedback for ongoing quality improvement.

### ED bypass

The effective management of STEMI patients begins before they reach the hospital. Encouraging individuals experiencing central chest pain to immediately call EMS (999/112) is a crucial first step, which can be facilitated through robust public education campaigns. This enables rapid pre-hospital assessment and direct patient transfer to a PCI-capable centre, thereby avoiding the delay associated with an unnecessary ED visit. This so-called ED “dwell time” is reported to be one of the most substantial and modifiable contributors to reperfusion delay, supporting the practice of ED bypass [10–15]. Encouragingly, the Irish Heart Attack Audit National Report for 2017–2020 reported an 81% rate of ED bypass, an improvement over previous years [16]. Nevertheless, the success of this approach hinges on the ability of EMS personnel to make informed decisions about patient routing. Despite protocols in place, 22 patients initially considered in our study were brought to the CHB ED despite having obtained a diagnostic ECG pre-hospital, either in the ambulance or a general practitioner’s office. As described in the Irish Heart Attack Audit National Report, key methods to facilitate ED bypass include ensuring emergency vehicles are equipped with 12-lead ECGs and that personnel are trained in its use and transmission of results, as well as the establishment of a “Code STEMI” freephone line to facilitate direct communication between EMS and the PCI centre [16].

### Study limitations

This study should be interpreted in the context of several limitations. Firstly, our study was limited to a single transferring centre and may not be generalizable to other institutions. Furthermore, our study focused on D2BT and its sub-intervals and did not examine other relevant outcomes such as total ischaemic time, mortality, or morbidity. Moreover, our study relied on retrospective analysis of existing data, which may be subject to human error and incomplete documentation. In particular, our analysis was restricted to the timepoints documented in the MMUH pPCI registry, limiting our ability to resolve various factors that may have contributed to delays, especially those occurring at the transferring hospital, making a strong case for the internal standardization of documentation at all transferring hospitals.

## Data Availability

The data used in this study are available upon request from the corresponding author, subject to any relevant privacy and confidentiality restrictions.

## Declarations

### Animal welfare

No animals were involved in this study.
